# Etiopathogenesis and diagnostics of autoimmune thyroid diseases

**DOI:** 10.1186/1756-6614-8-S1-A26

**Published:** 2015-06-22

**Authors:** Anhelli Syrenicz

**Affiliations:** 1 Department of Endocrinology, Metabolic and Internal Diseases Pomeranian Medical University in Szczecin, Poland

## 

The pathogenesis of Graves-Basedow's disease is similar to that of Hashimoto’s disease (chronic autoimmune thyroiditis) and it is assumed that these two forms are the opposite poles of the same autoimmune disease. The name of the first disease derives from the surnames of two scientists who described it in the literature: Robert James Graves (1835) and Karl Adolf von Basedow (1840). The first description of the second disease was presented in 1912 by Hakaru Hashimoto. Genetic, environmental and endogenic factors are involved in the pathogenesis of autoimmune diseases. Genetic predisposition to an autoimmune disease depends on many genetic loci, of which three seem to be most important: alleles of the major histocompatibility system genes (human leukocyte antigen – HLA), mainly of class II HLA-DR3 and DR5; alleles of the gene encoding antigen-4 associated with the cytotoxic T lymphocyte (cytotoxic T lymphocyte associated antigen-4 – CTLA-4) and alleles of the gene encoding the lymphocytic tyrosine phosphatase (Protein Tyrosine Phosphatase Non Receptor – Type 22 - PTPN22). Epidemiological studies also confirm that genetic factors are responsible for autoimmune thyroid diseases with a high prevalence in patients’ relatives, especially among monozygotic twins. The environmental factors responsible for the presence of thyroid autoantigens on thyrocyte surface include viral infection, extreme stress, an excessive iodine content in diet, the action of some drugs (interferon α, amiodarone) and a huge number of chemical compounds in polluted surroundings. The most important endogenic factors include female sex and the phenomenon of foetal microchimerism in pregnant women. An organ-specific suppressor T lymphocyte defect develops in genetically predisposed people, which leads to an excessive stimulation of the T-helper lymphocytes. As a consequence, B lymphocytes experience excessive stimulation with subsequent production of plasma cells, which are responsible for the disproportionate production of antibodies against thyroid antigens – mainly the TSH receptor, thyroglobulin and thyroid peroxidase. In Graves-Basedow's disease, TSH receptor is the main autoantigen against which the antithyroid antibodies are directed. High titres of ATG and anti-TPO may suggest the coexistence of Hashimoto’s disease. In 80% cases of Graves-Basedow's disease, TSHR antibodies are stimulatory (thyroid stimulating immunoglobulins – TSI). In some cases, antibodies that inhibit thyrotropin binding to thyroid cells are produced (thyrotropin binding inhibiting immunoglobulins – TBII), which may lead to the development of hypothyroidism.

In Hashimoto’s disease, cytotoxic lymphocytes, able to destroy thyroid follicular cells, receive considerable stimulation. Apart from cytotoxic lymphocytes, macrophages and the phenomenon of programmed cell death (apoptosis) also take part in the process of thyrocyte destruction. Clinical diagnosis of Graves-Basedow's disease is based on the detection of evenly enlarged thyroid gland with tactile fremitus and/or audible vascular murmur and the occurrence of the typical ophthalmic changes. Some patients also suffer from pretibial myxoedema and thyroid acropathy. Ultrasonography is a very useful imaging tool in the diagnosis of Graves-Basedow's disease – the thyroid gland is usually enlarged and has a homogenous, hypoechoic structure. Scintigraphy is not obligatory in each case of Graves-Basedow's disease, but it is very helpful in patients with the features of nodular structure and in the form without the goitre in which diagnostic difficulties occur. In the classical form of Hashimoto’s disease, a painless goitre of intensified cohesion presents with a butterfly-like shape because of the palpable pyramidal lobe. Patients complain of pressure and feeling of obstruction within the neck, and can report swallowing disorders and hoarseness. Chronic autoimmune thyroiditis can also occur in the atrophic, focal and juvenile form. Additionally, there are two variants of Hashimoto’s disease: postpartum thyroiditis and silent, painless thyroiditis. High anti-TPO antibody concentrations confirm the diagnosis of Hashimoto’s disease or any of its variants. The absence of antibodies in serum does not exclude lymphocytic thyroiditis as they are provided by intrathyroid lymphocyte infiltrations. Some authors attribute a special diagnostic significance to fine-needle aspiration biopsy in seronegative forms. Ultrasound thyroid examination is helpful in establishing correct diagnosis. Heterogeneous and distinctly hypoechoic structures of the thyroid gland are typical sonographic manifestations.

